# Joint Secondary Transcriptomic Analysis of Non-Hodgkin’s B-Cell Lymphomas Predicts Reliance on Pathways Associated with the Extracellular Matrix and Robust Diagnostic Biomarkers

**DOI:** 10.26502/jbsb.5107040

**Published:** 2022-09-27

**Authors:** Naomi Rapier-Sharman, Jeffrey Clancy, Brett E Pickett

**Affiliations:** 1 Department of Microbiology and Molecular Biology, Brigham Young University, Provo, UT 84602, USA

**Keywords:** B-cell, B-cell Non-Hodgkin Lymphoma, Biomarkers, Joint Analysis, Liquid Biopsy, RNA-seq, Transcriptomics

## Abstract

Approximately 450,000 cases of Non-Hodgkin’s lymphoma are annually diagnosed worldwide, resulting in ~240,000 deaths. An augmented understanding of the common mechanisms of pathology among larger numbers of B-cell Non-Hodgkin’s Lymphoma (BCNHL) patients is sorely needed. We consequently performed a large joint secondary transcriptomic analysis of the available BCNHL RNA-sequencing projects from GEO, consisting of 322 relevant samples across ten distinct public studies, to find common underlying mechanisms and biomarkers across multiple BCNHL subtypes and patient subpopulations; limitations may include lack of diversity in certain ethnicities and age groups and limited clinical subtype diversity due to sample availability. We found ~10,400 significant differentially expressed genes (FDR-adjusted p-value < 0.05) and 33 significantly modulated pathways (Bonferroni-adjusted p-value < 0.05) when comparing BCNHL samples to non-diseased B-cell samples. Our findings included a significant class of proteoglycans not previously associated with lymphomas as well as significant modulation of genes that code for extracellular matrix-associated proteins. Our drug repurposing analysis predicted new candidates for repurposed drugs including ocriplasmin and collagenase. We also used a machine learning approach to identify robust BCNHL biomarkers that include YES1, FERMT2, and FAM98B, which have not previously been associated with BCNHL in the literature, but together provide ~99.9% combined specificity and sensitivity for differentiating lymphoma cells from healthy B-cells based on measurement of transcript expression levels in B-cells. This analysis supports past findings and validates existing knowledge while providing novel insights into the inner workings and mechanisms of transformed B-cell lymphomas that could give rise to improved diagnostics and/or therapeutics.

## 1. Introduction

Lymphomas are the most common blood cancer, which primarily affects lymphocytes. There are three primary categories of lymphomas including Chronic Lymphocytic Leukemia/Small Lymphocytic Lymphoma, Hodgkin Lymphoma, and Non-Hodgkin Lymphoma. There are over 90 recognized types of Non-Hodgkin Lymphoma, which is diagnosed in ~450,000 patients worldwide annually, resulting in 240,000 deaths [1]. Among Non-Hodgkin lymphomas, only ~10-15% are T-cell lymphomas, while the remaining 85-90% are B-cell malignancies [2]. B-cell Non-Hodgkin Lymphomas (BCNHLs) pose a significant disease burden worldwide. BCNHL subtypes include Burkitt’s lymphoma, marginal-zone B-cell lymphomas, follicular lymphoma, diffuse large B-cell lymphoma, and mantle cell lymphoma [2]. B-cell lymphomas are dependent on their extracellular environment for activation and transformation into malignancies, including antigen activation of the B-cell receptor, canonical B-cell growth signals which are also essential to the maturation of healthy B cells, and signals delivered by other immune cells in the follicular/germinal center lymphoma microenvironment [3]. The research community has dedicated substantial effort to identify the attributes that characterize cancers across all types and subtypes—regardless of which tissue type first produces malignancies. Specifically, it has been suggested previously that all cancers share the following traits: selective proliferative advantage, altered stress response, vascularization, invasion and metastasis, metabolic rewiring, immune modulation, and an abetting microenvironment [4,5]. One example of a molecular mechanism that is common in cancer is malignant development through TP53 mutation, with multiple mutations in the TP53 being associated with hundreds of cancer subtypes [6]. Though not every gene-mechanism pairing will be widely found across malignant cells like TP53, identifying shared genes and mechanisms by performing joint secondary analysis on combined data from multiple previous research studies in a focused set of related cancer subtypes can be beneficial [7]. We can therefore leverage known mechanisms from well-studied subtypes to enable quicker, less expensive mechanism discovery for understudied subtypes. This approach could potentially enable researchers to identify shared mechanisms repurpose existing therapeutics to a wider swath of cancer types and subtypes. The widespread adoption of RNA-sequencing (RNA-seq) has opened new frontiers in disease research. Rather than identifying and characterizing individual cellular components, transcriptomic analyses can provide a mechanistic snapshot of the many genes that are upregulated or downregulated in response to a given stimulus or disease state, such as lymphoma. Characterizing these transcriptional patterns can aid in the identification of genes that could be worth further experimental investigation due to their selective modulation in diseased samples. Though the RNA-sequencing samples in the current study were previously published, analyzing them together in a joint secondary analysis can grant us new insights into disease mechanisms by increasing the signal of significant genes and reducing the statistical “noise” caused by outliers across patient subpopulations. The aim of this study was to perform a joint secondary analysis of transcriptomic data from de-identified publicly available B-cell Non-Hodgkin’s Lymphomas (BCNHLs) clinical samples to determine the shared underlying molecular mechanisms and biomarkers of B-cell lymphomas that are detectable after cellular transformation. We expect our analysis to validate past findings of B-cell cancer mechanisms and uncover mechanisms that have not been previously associated with BCNHL.

## 2. Methods

### 2.1 Collecting Samples

**Figure 1:**
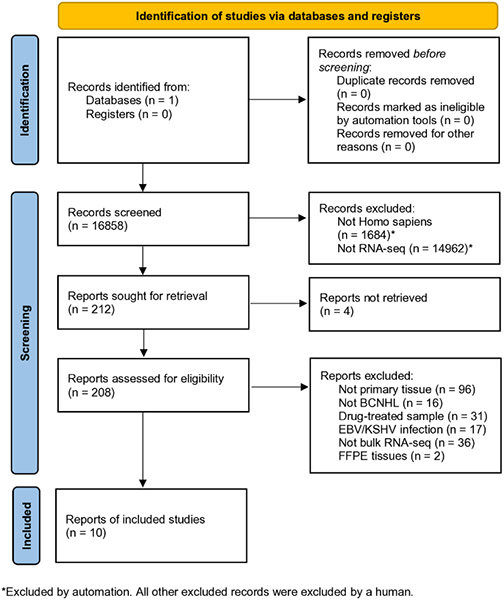
PRISMA flow diagram for transparent reporting of joint secondary analysis study selection. Contains a study-by-study breakdown of selection criteria. All studies included were retrieved from the Gene Expression Omnibus (GEO) database provided by NCBI.

RNA-sequencing samples were acquired from the National Center for Biotechnology Information (NCBI) Gene Expression Omnibus (GEO) database [8] using the search term, “b-cell lymphoma” with the goal of finding B-cell non-Hodgkin’s lymphoma samples and healthy B-cell controls. The automatic GEO filters “Homo sapiens” and “high-throughput RNA-sequencing” were applied. Cell lines, formalin-fixed paraffin-embedded tissues, gene expression microarray experiments, single-cell (10X) RNA-sequencing experiments, xenografts, samples known to be infected with EBV and KSHV, and samples which contained more diverse cell types (i.e., whole blood, lymph node, PBMCs, brain, etc.) were excluded by hand. All samples that had one or more of these disqualifying attributes were excluded from the dataset prior to analysis, meaning that only a subset of the samples from an individual experiment may be represented in the joint secondary analysis. One study which matched all criteria was excluded due to inconsistent and unreliable sample labeling. Multiple myeloma, leukemia, and Hodgkin’s lymphoma samples were intentionally excluded in favor of focusing on B-cell non-Hodgkin’s lymphomas. Records were passed or failed against the standardized exclusion criteria detailed above by one team member, with some input from a second team member. To avoid inclusion bias, any sample that could not be excluded by our standardized exclusion criteria was included in the study. While a subset of the healthy control samples was obtained from the same RNA sequencing projects as the BCNHL samples, others were obtained from three lymphoma-unrelated B-cell datasets with healthy controls to create roughly equivalent-sized BCNHL and healthy groups. Final dataset assembly from GEO concluded on October 22, 2020, resulting in a dataset of 322 samples (134 BCNHL samples and 188 healthy B-cell controls) from ten studies [9-20]. The raw data for these experiments were previously collected by the primary authors and conform to the appropriate ethical oversight to protect patient autonomy and patient identity. All 10 primary RNA-sequencing datasets from which we gathered samples for our lymphoma joint secondary analysis have been published in the peer-reviewed literature, increasing overall confidence that each dataset has acceptable quality (Table 1, Figure 1).

### 2.2 Preprocessing of RNA-Sequencing Data

Following the manual curation of the RNA-seq samples, the fastq files were pre-processed as previously described [21]. In brief, fastq files containing RNA-sequencing data were downloaded from the Sequence Read Archive (SRA) using the sratools software package. The fastq files, the associated metadata file, and a configuration file for each dataset were then generated and used as input to the Automated Reproducible MOdular workflow for preprocessing and differential analysis of RNA-seq data (ARMOR) workflow [22]. A configuration file was used by ARMOR to appropriately set up a python-based Snakemake workflow [23]. In the ARMOR workflow, adapters and poor-quality regions of reads were trimmed with TrimGalore! [24], quality control metrics were calculated with FastQC [25], reads were mapped to the human GRCh38 transcriptome and total gene transcripts quantified with Salmon [26], significant differential gene expression was calculated using a negative binomial distribution implemented in edgeR [27], Gene Ontology (GO) enrichment was performed against terms from the MSigDB [28] while adjusting for inter-gene correlation using the Camera algorithm [29], and significant splice variants were predicted with DRIMseq [30]. The significant differentially expressed genes from the ARMOR workflow were then used as input to the signaling pathway impact analysis (SPIA) algorithm to enrich differentially expressed genes against intracellular signaling pathways from five databases including KEGG, Panther, BioCarta, Reactome, and NCI [31-35]. Differentially expressed genes outputted by ARMOR and DRIMSeq were evaluated by the effect measures log_2_ fold change and likelihood ratio respectively. Confidence in results was accomplished using false discovery-rate adjusted p-values.

### 2.3 Additional Analysis and Visualization of Differentially Expressed Genes and Gene Ontologies

**Figure 2:**
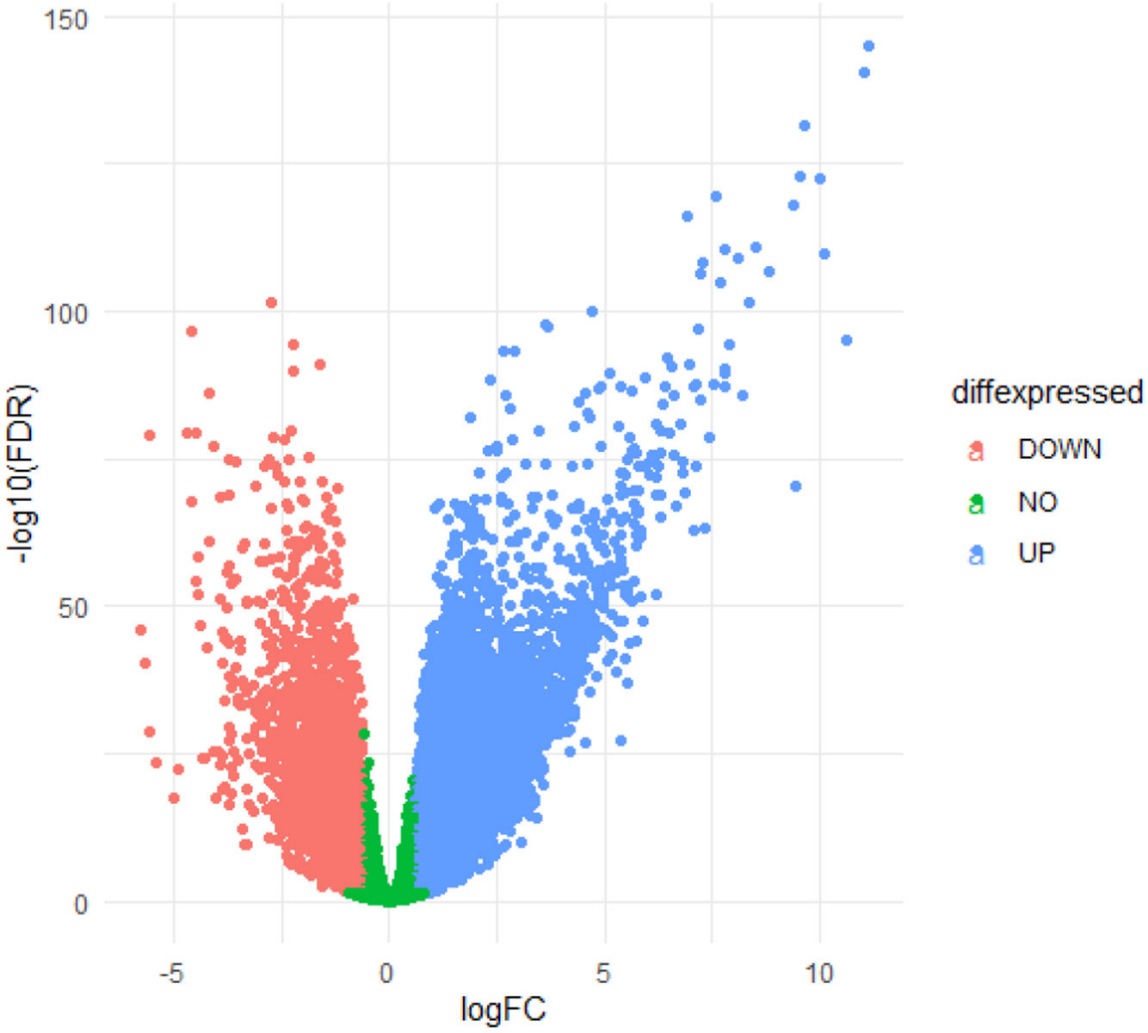
Visualization of Differentially Expressed Genes and Gene Ontologies. Differentially expressed gene volcano plot. Green dots represent genes which were not significantly differentially expressed between healthy B-cells and BCNHL, while the salmon and blue dots represent downregulated and upregulated genes respectively.

**Figure 3:**
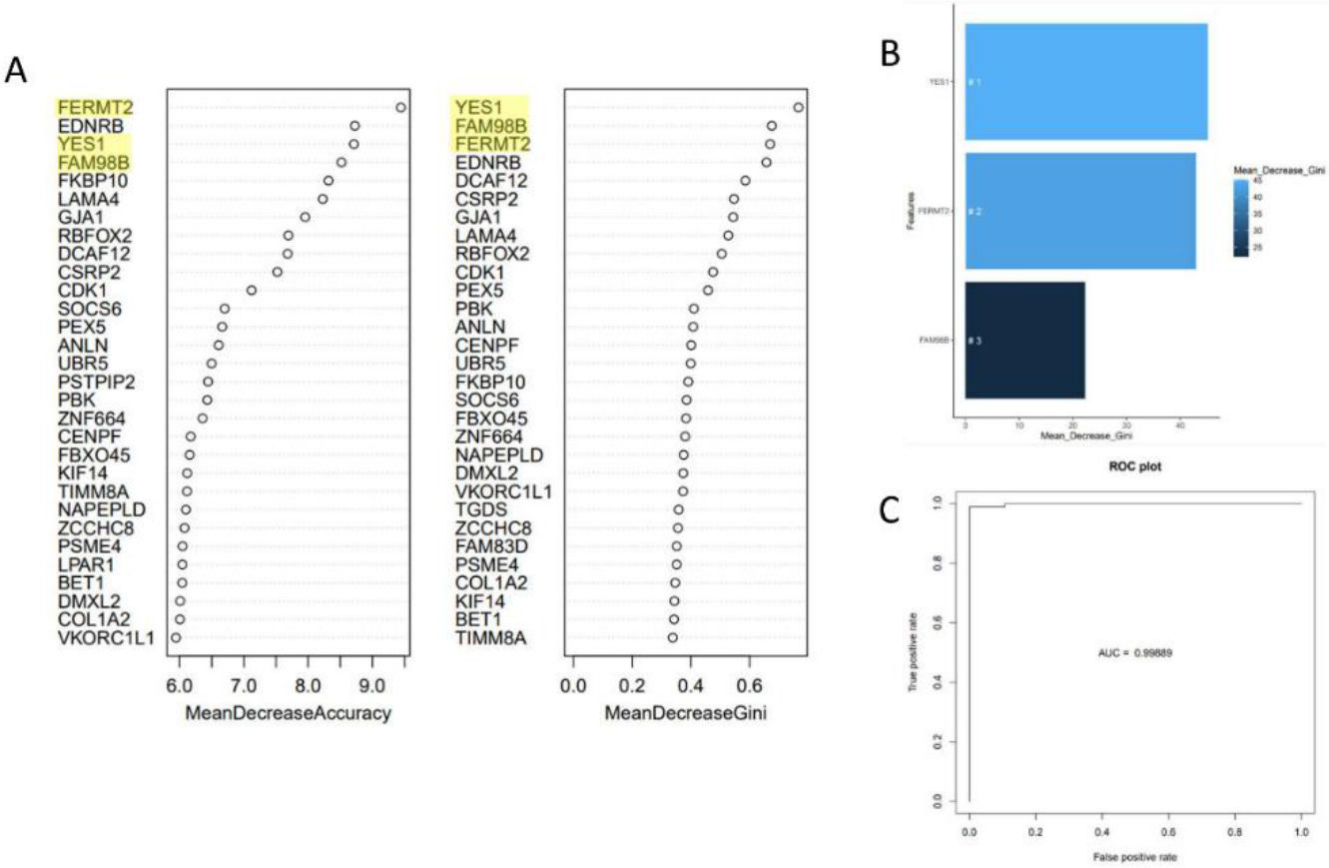
Biomarker Prediction Yields a Three-Gene Signature with 99% Predictive Ability. Random forest analysis was conducted using the normalized read counts for all sequenced genes from each sample and the disease condition associated with each sample (healthy or lymphoma). A) Initial random forest biomarker results quantified with mean decrease in Gini impurity and mean decrease in permutation show YES1, FAM98B, and FERMT2 as the highest-ranked diagnostic biomarkers (ranked by mean decrease of Gini impurity score). B) Random forest results for the top three genes in isolation. C) Receiver-operator characteristic curve using only YES1, FAM98B, and FERMT2 shows these three genes have 99.889% specificity and sensitivity when predicting BCNHL status (healthy or diseased) based on B-cell transcripts.

The PRISMA flowchart template was used to generate figure 1, which is consistent with the accepted transparent reporting of joint secondary analysis generation and results [36]. The R package ggplot was used to construct the Fig 2 volcano plot from using FDRs and log_2_ fold change values for each gene from the edgeR output [37]. The KEGG ontology was extracted from the Brite Hierarchy using existing code [32]. Genes included in the Brite Hierarchy were then computationally matched to their corresponding edgeR log_2_ fold change values. A statistical enrichment of the KEGG gene ontologies was performed using the R package bc3net [38] prior to visualizing the bc3net enrichment results with the R package Treemap in Fig 3 [39].

### 2.4 Biomarker Prediction using Differentially Expressed Gene Data

Transcript-level read counts, generated by Salmon, were organized into a tabular format and samples were randomly assigned to either the testing set (30%) or the training set (70%). The R package randomForest was used to run a supervised classification analysis, with disease state (healthy or lymphoma) as the predictor, to determine biomarkers [40]. The initial results from the whole transcriptome were then reduced to the 3, 5, and 10 best-scoring transcriptional biomarkers, based on the mean Gini impurity decrease values for each of the features. These values were then sorted by size to determine the transcribed genes from the original dataset with the largest association. The area under the curve (AUC) was calculated from the receiver operator characteristic curves that were generated for each set of random forest results to determine the efficacy of the selected biomarkers for disease prediction.

### 2.5 Drug Prediction using Differentially Modulated Pathways

Drug prediction was conducted using the Pathways2Targets2.R algorithm [41]. Significantly modulated pathways (as determined by SPIA) were used as input for the Pathways2Targets algorithm to determine existing drugs that could potentially be repurposed for BCNHL. The Pathways2Targets algorithm takes the significantly affected pathways determined by SPIA, finds the members of those pathways, and searches the Open Targets drug database for drugs known to target the proteins from each pathway. The output table from this process was then summarized using a custom R script, most_common_treatments_2021_09_19.R [42].

## 3. Results

We acquired our BCNHL samples from publicly available projects on the NCBI Gene Expression Omnibus (GEO) database using the search term, “b-cell lymphoma” with the goal of finding B-cell non-Hodgkin’s lymphoma samples and healthy B-cell controls [8,21]. We excluded non-human samples, cell lines, formalin-fixed paraffin-embedded tissues, gene expression microarray experiments, single-cell (10X) RNA-sequencing experiments, xenografts, samples known to be infected with EBV and KSHV, and samples which contained more diverse cell types (i.e., whole blood, lymph node, PBMCs, brain, etc.). Study GSE142334 matched all study criteria, but contained file types incompatible with our bioinformatic workflow and was excluded due to inaccessibility. Study GSE93627, which seemed to match all of our criteria, was excluded in later stages of selection due to inconsistent and unreliable sample labeling. We also intentionally excluded multiple myeloma, leukemia, and Hodgkin lymphoma samples in favor of focusing on B-cell non-Hodgkin lymphomas. We then located additional healthy B-cell control samples from BCNHL-unrelated studies to even out case and control numbers, the final three studies cited in Table 1. In an effort to conform to best-practice PRISMA guidelines on transparent reporting of secondary joint analyses [36], we have included a detailed diagram on our sample selection process and PRISMA’s transparent reporting checklist (Fig 1, S1 File). Our final dataset included a total of 322 samples (134 BCNHL samples and 188 healthy B-cell controls) from ten studies (Table 1) [9-20]. Though the samples included in our joint secondary analysis were all clinical samples, the metadata provided by each original project varied greatly in both quantity and nature, making it difficult to discern the extent of sample heterogeneity or homogeny for variables other than lymphoma subtype. Given that the aim of this study was to generate a mechanistic profile for many BCNHL samples in comparison to healthy B-cells, the only evident source of heterogeneity is the distribution of BCNHL types across the included samples. We recognize that the included samples were largely skewed toward the diffuse large B-cell lymphoma subtype, which is the most common BCNHL subtype and resultantly has more data available on GEO than any other BCNHL subtype.

We began by trimming, mapping, and quantifying the RNA-sequencing reads prior to calculating the significant differential gene expression when comparing the Lymphoma samples to the non-diseased control samples. This comparison returned ~13,800 significant differentially expressed genes (DEGs) (Fig 2, Table 2, S2 and S3 Files). We then ranked this list by the FDR-corrected p-value for each gene. We observed that the top 20 DEGs include both novel and accepted differentially expressed genes associated with various Lymphomas. Specifically, we confirmed several genes that have previously been explored or characterized in various subtypes of BCNHL including Apolipoprotein C1 (APOC1; log_2_FC = 6.93, FDR = 8.55 × 10^−117^) and Vascular cell adhesion molecule 1 (VCAM1; log_2_FC = 7.85, FDR = 2.29 × 10^−120^) to be upregulated in BCNHLs. We also found two pathological BCNHL genes, C-C motif chemokine ligand 18 (CCL18; log_2_FC = 10, FDR = 3.74 × 10^−123^) and C-X-C motif chemokine ligand 9 (CXCL9; log_2_FC = 11, FDR = 4.31 × 10^−141^) to be upregulated in BCNHL as compared to healthy B-cells.

We then examined the highest-ranking novel differentially expressed genes from our joint secondary analysis to identify transcriptional mechanisms of disease, with transcripts reported at the gene level. The first gene we observed using this approach was Lumican (LUM), which is a member of the small leucine-rich proteoglycan family (SLRPs) [43], and was substantially upregulated in lymphoma (log_2_ fold change = 11.1, FDR p-value = 1.11 × 10^−145^). In addition, the larger family of SLRPs appears to play a role in BCNHL (Table 3). Specifically, our data show that 12/18 SLRPs are expressed in healthy and/or cancerous B-cells, and that 11/12 B-cell-expressed SLRPs are significantly differentially expressed in BCNHL samples. We found that overall, the SLRP fold changes substantially differed (9/12 expressed SLRPs are upregulated, 2/12 are downregulated, 1/12 had no significant change), with the genes encoding SLRPs (especially Classes I and V) being well represented in the B-cell lymphoma transcriptome.

We found that genes encoding the Complement C1q A (C1QA; log_2_FC = 9.54, FDR = 2.03 × 10^−123^), Complement C1q B (C1QB; log_2_FC = 9.4, FDR = 8.19 × 10^−119^) and Complement C1q C (C1QC; log_2_FC = 9.65, FDR = 2.7 × 10^−132^) chains were all dramatically and significantly upregulated in BCNHL. Complement proteins are typically regarded as components of the innate immune system, which bind to antigen-antibody complexes to facilitate the formation of the membrane attack complex to kill invading bacteria. Our finding adds to the growing body of work indicating an association between complement C1q expression and lymphoma pathology. Additionally, we detected AL512646.1 (also known as LOC100128906 and as a WDR45-like pseudogene) as differentially expressed by B-cell non-Hodgkin’s lymphoma samples, a novel observation which was somewhat unexpected. Though AL512646.1 is annotated as a pseudogene and has not been previously associated with cancer, the RNA-sequencing data shows that it is uniformly expressed in healthy B-cells and downregulated in at least a subset of BCNHLs (log_2_FC = −15.1, FDR = 2.24 × 10^−115^). Next, we used the DRIMSeq algorithm to determine which genes had significant differences in the presence of splice variants between BCNHL and healthy control samples. This analysis returned 320 genes for which splice variants were significantly different (Table 4, S4 File). Apolipoprotein E (APOE) was the most statistically significant splice variant (Lr [likelihood ratio] = 4470, # of alternate splice variants = 4, adjusted p-value = 0). Specifically, we observed the expression of APOE transcripts ENST00000252486, ENST00000425718, ENST00000434152, ENST00000446996, and ENST00000485628 to significantly differ between non-Hodgkin’s lymphoma and non-diseased B-cells.

We also observed that Collagen type I alpha 1 chain (COL1A1) had significant splice variants (Lr = 1520, # of alternate splice variants = 12, adjusted p-value = 5.55999999807983 × 10^−315^). Interestingly, our study also found that the COL1A1 gene was significantly upregulated in BCNHL (log_2_FC = 3.73, FDR = 9.78 × 10^−48^). We also observed novel significant splice variants in Collagen type XXVII alpha 1 chain (COL27A1), which was found to be significant in BCNHL (Lr = 1060, # of alternate splice variants = 7, adjusted p-value = 6.71 × 10^−220^). We then wanted to determine which functional terms in the Gene Ontology (GO) were over-represented by the list of DEGs in BCNHL. The Camera algorithm evaluated 14,901 terms (including gene ontologies and human phenotypes) for statistical enrichment against the significant differentially expressed genes that we generated with edgeR. Although there were 482 results (p-value < 0.05), none remained significant after multiple hypothesis correction (S5 File). The lack of significant results is somewhat expected given the overall molecular heterogeneity of BCNHL subtypes and the stringency of the Camera algorithm. To visualize the Gene Ontology changes, we used a hypergeometric enrichment algorithm that applied a p-value cutoff of 0.05. We then averaged the edgeR fold-change values for the genes of each gene ontology in the KEGG Brite hierarchy and plotted the enrichment results using the R Treemap package to better understand the contribution of various GO terms to the overall list of DEGs (S3 File). To better understand the results of our analysis at a more mechanistic level, we used the signaling pathway impact analysis (SPIA) algorithm to identify intracellular signaling pathways that play important roles in various subtypes of lymphoma after transformation. Briefly, this pathway-analysis algorithm generates a null distribution through bootstrapping to identify pathways that are significantly modulated based on the DEGs. Our analysis revealed 33 significantly modulated pathways between lymphoma B-cells and non-diseased B-cells (Table 5, S6 File). Specifically, we observed ten pathways that were involved with the extracellular matrix and connective tissue, bolded below in Table 5. The upregulation of these pathways indicates that transformed BCNHL likely benefits from modulations to the extracellular matrix.

We next used the Pathways2Targets algorithm to identify potentially novel drug targets for BCNHL from the signaling pathway results (S7 File). *De novo* drug development can require decades and billions of dollars, whereas drug repurposing, which is defined as finding new indications for existing drugs, is much cheaper and faster. Many existing drugs have undergone in-depth research to identify their target proteins, and this target information is stored in databases such as DrugBank and OpenTargets. In brief, the Pathways2Targets algorithm takes the significant pathways (as previously determined by SPIA), finds each protein member of those pathways, and searches the OpenTargets database [6] for all drugs known to directly interact with each protein, and generates an extensive table containing all drugs known to interact with protein members of the significant pathways (S8 File). We sorted the results so that drug targets present in multiple signaling pathways would be ranked higher (Table 6). Though Pathways2Targets results are in no way conclusive of drug efficacy for a novel indication, the algorithm provides a short-list of drugs for subsequent validation in the laboratory and has a track record of returning many drugs already in use for a given disease and several novel drug candidates [44-47]. Based on the Pathway2Targets output, we predicted the most relevant existing FDA-approved drugs for other indications that could affect the lymphoma phenotype are Doxycycline, Ocriplasmin, and Collagenase. We also identified ATN-161 as a candidate drug, but it has only been tested in phase-two trials. Doxycycline is currently in use for BCNHL subtypes [48]. The other drug candidates are promising based on drug targeting data but require follow-up validation experiments.

Rather than solely rely on the significant differential expression data to determine biomarkers, we applied a more robust random forest machine learning classification method to predict biomarkers of BCNHLs. Specifically, the DEG statistics focus on identifying genes that have a significantly large difference in expression between two states (lymphoma vs. healthy), while the random forest approach identifies genes that consistently show distinct profiles that are strongly dependent on disease state (lymphoma vs. healthy). Consequently, the random forest approach generates a model to identify gene products that are capable of most accurately classifying either disease or healthy states for the data analyzed. The top three genes identified by our random forest analysis included YES1, FAM98B, and FERMT2 (Table 7, Figs 3A and 3B, S9 File). We then calculated the area under the curve for the receiver-operator characteristic curve, which showed that when the expression values from these three genes have a combined specificity and sensitivity of 99.889% when classifying whether the patient samples had BCNHL (Fig 3C). The top three genes identified by our random forest biomarker prediction are high-fidelity biomarkers of BCNHL due to their consistent and extreme upregulation across our 134 clinical BCNHL samples as compared to our 188 healthy B-cell samples. YES proto-oncogene 1, Src family tyrosine kinase (YES1), FERM domain containing kindlin 2 (FERMT2), and family with sequence similarity 98 member B (FAM98B) have previous associations with cancers, but our finding that they are associated with BCNHL as high-potential biomarkers is novel.

## 4. Discussion

The goal of this study was to collect and analyze publicly available RNA-seq data from GEO to find differentially expressed genes, pathways, splice variants, and biomarkers that are relevant to BCNHL after the cells are initially transformed. We confirmed several biologically- and clinically-relevant biomarkers and pathologic mechanisms that were identified previously, as well as novel entities. We found several key genes that are significantly differentially expressed in BCNHL including LUM and other SLRPs, complement protein components, and the supposed pseudogene AL512646.1. We confirmed that previously characterized biomarkers such as APOC1, VCAM1, CCL18, and CXCL9 are overexpressed in BCNHL, and that 320 genes including APOE, COL1A1, and COL27A1 had differentially expressed splice variants. We additionally found a BCNHL reliance on the upregulation of pathways associated with the extracellular matrix. We also predicted three transcriptional biomarkers that perform well at differentiating patients who have BCNHL from those who do not. To our knowledge, this is the largest joint secondary transcriptomic analysis of primary human samples in the BCNHL field to-date. Two large-scale integrative multi-platform genomic profiling projects were previously completed on diffuse large B-cell lymphoma (DLBCL) with the aid of transcriptomic sequencing [49, 50]. However, their applications of the RNA-sequencing data are distinct from the approach and purpose reflected in this project. Specifically, these prior studies used RNA-seq data to identify causative gene fusions as well as to predict subtypes, and to determine the location and frequency (respectively) of genetic aberrations that initiate disease. In contrast, we utilized RNA-seq data to better characterize the gene expression profiles of BCNHL after transformation had occurred, which provides a high-level view of characteristics that are shared across multiple BCNHL types. Though our applications of RNA-seq data were distinct, we were interested to find some shared results. Reddy *et al.* found that extracellular matrix and lymphatic vessel gene sets were important differentiators between their 33 gene expression-based proposed subtypes [49], while we noted an overall trend of extracellular matrix-associated pathway upregulation. Schmitz *et al.* noted the gain-of-function of multiple crucial genes along the PI3K pathway [50], which seems consistent with our data where we found two PI3K pathways to be upregulated. We believe that including representative samples from multiple BCNHL subtypes augments the signal(s) that are shared among the represented subtypes and could aid in the identification of shared mechanistic insights with reduced bias. Given our intentional focus on BCNHL, we did not include multiple myelomas, B-cell leukemias, or Hodgkin’s B-cell lymphomas. Promising future directions may include querying multiple databases for sequencing data and perhaps expanding the scope of future joint secondary analysis to include all B-cell malignancies. Since we used only publicly available data, there may be biases in age, gender, or ethnicity. Though there is previous evidence in the literature that directly associates BCNHL with some of our results, some of our findings are novel to BCNHL. We will therefore appeal to other models in cancer (i.e., other blood cancers, other non-blood cancers) in cases where no previously published research indicates the relationship between BCNHL and our results. Comparing our results against those from cancers outside of the BCNHL family is a direct appeal to the Hallmarks of Cancer [4]. Given that underlying mechanisms for cell growth, vascularization, disruption of the cell cycle, and other cellular attributes have the potential to be common across cancer subtypes, we expect that including research from different cancer models will help to accelerate research into shared cancer mechanisms. We therefore first pull on any research available in BCNHL, followed by research in other B-cell malignancies, other blood cancers, and finally all other cancers. We believe that identifying a possible mechanism for a gene that is associated with other cancers, but unresearched in BCNHL, is still relevant. We expect that a subset of these findings will justify additional wet lab experimentation.

### 4.1 Differentially Expressed Genes Suggest Shared Underlying Mechanisms for Lymphomas

Lumican (LUM) seems to play a role in the progression or non-progression of several different cancer types. Mahadevan *et al.* previously reported upregulated LUM in both T- and B-cell lymphomas, but offered no insights on potential mechanisms [51]. A literature search of parallel systems revealed that in breast cancer, high stromal-cell expression of LUM adjacent to the tumor stalls tumor growth, and lowered stromal expression of LUM correlates with higher breast cancer mortality rates and increased severity [52]. In melanoma, LUM in the extracellular matrix halts metastasis through direct interaction with alpha-2-beta-1 integrin [53]. Both breast cancer and pancreatic cancer cells have been documented to upregulate LUM, along with many other cancer types [43]. Overall, LUM expression by cancer cells seems to correlate with more aggressive cancers and poorer patient outcomes. The massive LUM upregulation illustrated in our samples may be because the BCNHL samples available on GEO were mostly from advanced or refractory cases of BCNHL. The prior finding that high LUM expression around tumors is protective against metastasis in several cancer subtypes indicates the potential for LUM as a cancer-stalling therapy. Interestingly, a subset of the members in the SLRP protein family have been previously identified in B-cell Non-Hodgkin’s lymphomas including DCN [54], BGN [54], ASPN [55], FMOD [56], LUM [51], PRELP [56], and TSKU [57]. However, other members within the SLRP family have not been previously considered as lymphoma biomarkers or potential pathology-inducing molecules. Our novel finding is that the SLRPs ECM2, CHAD, PODN, and PODNL1 are differentially expressed in BCNHL. Proteoglycans have been shown to be associated with pro-cancer mechanisms in prostate, breast, colon, lung, ovary, mesothelium, pancreatic, lymphoma, and esophageal cancers [43]. Our results show two upregulated pathways in BCNHL that were previously shown to be mechanistically intertwined with proteoglycans in cancer, which are the Focal Adhesion pathway [58] and the PI3K-Akt signaling pathway [59]. Taken together, these data may suggest a connection between previously established proteoglycan cancer mechanisms and B-cell non-Hodgkin’s lymphomas. Additional work is still required to elucidate the role(s) that these entities play in BCNHL.

Discussing our other top DEGs in the context of other cancers, increased expression of complement genes C1QA and C1QB at week 16 of mantle cell lymphoma treatment by Venetoclax and Ibrutinib was significantly associated with a worse prognosis [60], illustrating that C1QA and C1QB may be associated with resistance to cancer drugs. Jiang et al. showed via immunohistochemistry that C1QB localizes to the nuclei of gastric cancer cells [61]. C1QB’s nuclear localization suggests that C1QB may have additional function(s). Upregulation of C1QA, C1QB, and C1QC in peripheral T-cell lymphoma [62] and upregulation of C1QC in Epstein-Barr Virus-positive diffuse large B-cell lymphoma [63] have been reported previously. Though it is possible that BCNHL is up-regulating expression of C1q chains A, B, and C in response to underlying patient-cohort deficiency in complement function, the whole C1q protein has been shown to mediate metastasis, motility, growth and proliferation, and adhesion in multiple other *in-vitro* and *in-vivo* cancer models [64].We consider this C1q research across multiple cancer types to indicate that an alternate, cancer-associated C1q function in BCNHL merits further investigation. Our results add to the growing body of work suggesting a potential alternate function of complement proteins in cancer that warrants further investigation.

In addition to our novel findings on differentially expressed genes, we were also able to detect statistically significant genes that were previously characterized in at least one subtype of BCNHL. The first of these proteins is Apolipoprotein C1 (APOC1), which we observed to be upregulated in BCNHL. APOC1 is one of three genes whose expression levels are predictive of diffuse large B-cell lymphoma severity [65], and it is also upregulated in late stage lung cancers as compared to early stage lung cancers [66]. This suggests that APOC1 may be contributing to cancer pathology across diverse cancers in multiple cell types. Our observation that C-C motif chemokine ligand 18 (CCL18), which has a well-recognized role in lymphoma, was upregulated in our BCNHL analysis is relevant since this gene assists large B-cell lymphoma in cell proliferation, the NF-Kappa-B pathway, and the PI3K-AKT pathway [67]. Its upregulation in macrophages and dendritic cells from cutaneous T-cell lymphoma lesions was associated with a negative prognosis [68]. Our finding of C-X-C motif chemokine ligand 9 (CXCL9) to be significantly upregulated in our analysis of B-cells is interesting since this gene has been shown to promote the progression of diffuse large B-cell lymphoma by halting degradation of beta-catenin (CTNNB1) and upregulating its initial expression [69]. Our findings support this proposed mechanism with CTNNB1 being upregulated in lymphoma (log_2_FC = 1.1, FDR = 1.54 × 10^−33^), while other elements of the CTNNB1 “destruction complex” were mostly downregulated. Specifically, several of the known components of the destruction complex that were detected in our analysis include APC (log_2_FC = −0.755, FDR = 3.51 × 10^−11^), GSK3B (log_2_FC = −0.692, FDR = 2.62 × 10^−3^), CSNK1A1 (not significant), AXIN1 (log_2_FC = 0.533, FDR = 3.96 × 10^−10^), BTRC (not significant), and FBW11 (log_2_FC = −0.692, FDR = 5.60 × 10^−20^). We identified several other genes that may be relevant to cancer pathology. Small but significant upregulation of AXIN1 is of interest for additional investigation due to its ties to CXCL9, and is not known to have multiple heterogenous functions [70]. AXIN1 regulates the Wnt and Janus Kinase (JNK) signaling pathways [71], and it regulates the Wnt pathway by degrading CTNNB1 [50]. If CTNNB1 is not degraded by AXIN1, CTNNB1 translocates to the nucleus and interacts with LEF1, which we found to be significantly upregulated, and TCF7 (not significant in this study), causing transcription of Wnt pathway target genes to occur [72, 73]. Wnt helps to regulate cell cycle and contributes to the increased growth rate of many cancer types [74]. AXIN1 activates the JNK signaling pathway by binding to MAP3K1, which we found to be significantly downregulated, or to MAP3K4, which was significantly upregulated [75]. Since CTNNB1 has been shown to contribute to apoptosis resistance in multiple myeloma cells [76], it is possible that BCNHL’s decreased ability to destroy CTNNB1 in may contribute to a similar pathogenic mechanism. Finally, VCAM1 upregulation is associated with a poor prognosis for patients with non-Hodgkin's lymphomas, and VCAM1 is under investigation as a potential serum biomarker for assessing disease progression [77]. Adhesion molecules such as VCAM1 promote cancer metastasis, or in the case of blood cancers, extravasation, by allowing cancer cells to exit the bloodstream and integrate with healthy tissues throughout the body [78].

### 4.2 Splice Variants Suggest Relevance to Lymphomas

To better understand the contribution of differentially expressed splice variants to disease, we examined the highest-ranked DRIMseq results. Our observation that Apolipoprotein E (APOE) was the highest-ranking splice variant result for BCNHL resonates with previous findings that associate this gene with pancreatic cancer pathology [79]. In addition, pediatric patients with malignant lymphoma and acute lymphoblastic leukemia who express isoforms E3 and E4 of APOE are at higher risk of developing extreme hypertriglyceridemia [80]. Though little research has been done concerning the mechanisms of APOE in BCNHL, we believe that APOE may be contributing to disease by participating in the Regulation of Insulin-like Growth Factor (IGF) activity by Insulin-like Growth Factor Binding Protein (IGFBP) Pathway, which is we found to be a significantly modulated pathway that includes APOE. The significance of APOC1 as a DEG in BCNHL, paired with the evidence of significant APOE splice variants suggest that apolipoproteins may be useful targets for future BCNHL treatments. Our observation of Collagen type I alpha 1 chain (COL1A1) as a highly ranked splice variant result is novel to the best of our knowledge. However, the literature indicates that the COLA1A-014 transcript regulates the CXCL12-CXCR4 axis in gastric cancer, leading to tumor progression [65]. In addition to displaying significant differences in splice variant expression, we also found COL1A1 to be significantly upregulated in BCNHL. COL1A1 has previously been reported to be upregulated in peripheral T-cell lymphoma [32]. In Hodgkin’s lymphoma, COL1A1 overexpression is associated with epigenetic silencing of the RNA demethylase ALKBH3 and reduced survival [81]. COL1A1 is a member of several of our significant upregulated pathways involving the extracellular matrix (ECM-receptor interaction, Focal adhesion, Extracellular matrix organization, and Collagen formation). This involvement in extracellular matrix-related pathways strengthens the case that the mechanism of COL1A1 may involve tumor cell interaction with its outer environment. Collagen type XXVII alpha 1 chain (COL27A1) having significant changes among its expressed splice variants in BCNHL is interesting since it was recently reported as being overexpressed in adenoid cystic carcinoma [82]. Like COL1A1, COL27A1 is a member of the upregulated Extracellular matrix organization and Collagen formation pathways, suggesting that COL27A1 could play a role in BCNHL extravasation.

### 4.3 Extracellular Matrix-Related Pathways may contribute to Disease

Our signaling pathway enrichment analysis broadened the scope of our analysis and interpretation. Many of our findings supported an interesting reliance of BCNHL on pathways associated with the extracellular matrix. Recent research has suggested the importance of extracellular matrix components in reactivating quiescent cancer cells through the β1-integrin signaling pathway [83]. It would follow that interaction with extracellular matrix components also plays a role in regulating cancer cells. To our knowledge, no studies have reported the integrin signaling pathway to be activated in BCNHL, though it has been reported as activated in the closely-related cancer NK/T-cell lymphoma [51]. The activation of these pathways suggests that malignant BCNHL cells may have an advantage by interacting with the extracellular matrix. Such interactions with the extracellular matrix are typically considered to be an important part of metastasis [78]. We found this result to be interesting since lymphomas are liquid tumors, unbound by extracellular matrix. This upregulation of pathways allowing interaction with the extracellular matrix may suggest that BCNHL could be invading non-lymphatic and/or non-circulatory tissues. The trend of extracellular matrix interaction is also seen in the DEG results, adding support to the idea that interaction with the extracellular matrix is important for BCNHL growth and survival. Additionally, COL1A1 and COL27A1, which are members of extracellular matrix-related pathways, are two of the genes with the most significantly differential expression of splice variants.

### 4.4 Drug Prediction Algorithm Returned both Tested and Novel Candidates

Of our top drug results, doxycycline is currently in use for ocular B-cell lymphomas [84, 85]. It is additionally under investigation for diffuse large B-cell lymphoma; recent work found doxycycline suppresses diffuse large B-cell lymphoma growth *in vitro* and *in vivo* via CSN5 inhibition [48]. ATN-161 is a novel drug candidate for BCNHL. Though it has only been tested in phase two of clinical trials, it has been a successful drug against refractory solid tumors, making it a promising drug candidate for other susceptible malignancies [86]. ATN-161 suppresses cancer via integrin beta1 alpha5 antagonism, disabling invasion and metastasis [87]. Ocriplasmin reverses vitreomacular adhesion via interaction with fibronectin and laminin [88]. Though ocriplasmin has never been used in cancer before, it may be a promising drug candidate due to its ability to modulate adhesion. Collagenase clostridium histolyticum is under investigation for treating collagen-rich uterine fibroids and was successful at reducing the stiffness of the tumors [89]. These predictions justify further validation experiments to determine their relevance in human BCNHL.

### 4.5 Machine Learning Predicts Novel Biomarkers of BCNHL

YES1, FERMT2, and FAM98B are novel biomarkers not previously associated with BCNHL. However, each has well-documented cancer associations. YES1 is a tyrosine kinase which regulates cell cycle and apoptosis *in vitro* and cell growth *in vivo* of tumors with YES1 amplification [90]. YES1 has been previously identified as a biomarker for non-small cell lung cancer and esophageal adenocarcinoma [91, 92] and may be a potential membrane biomarker. YES1 can anchor to the inner membrane with help from peptide SMIM30 [93], but whether it can flip to the outer leaflet has not been investigated. The role of YES1 in BCNHL pathology also needs additional investigation. FERMT2 has been pinpointed as a biomarker for other cancers previously including non-small cell lung cancer and prostate cancer [94, 95], but not for BCNHL. FERMT2 stabilizes CTNNB1, which is a well-documented activator of oncogene transcription, and is implicated in Wnt pathway regulation [96]. Additionally, FERMT2 enhances integrin signaling and mediates migration, invasion, and focal adhesion [97, 98]. Though FAM98B has been shown to play an important role in the development of multiple cancers, it has not previously been identified as a biomarker for any cancer. FAM98B is an arginine methyltransferase utilized in tumorigenesis and works in tandem with DDX1, a pan-cancer marker, in RNA metabolism/processing [7, 80]. Like YES1 and FERMT2, FAM98B has not been previously identified as a biomarker for BCNHL. These three genes have substantial diagnostic potential as a liquid biopsy that could be generalizable across B-cell non-Hodgkin’s lymphoma subtypes. Further experimental validation is needed to determine whether these are suitable as diagnostic or prognostic biomarkers.

## 5. Conclusions

In summary, our joint secondary analysis identified many significant differentially expressed genes and pathways that play a role in B-cell non-Hodgkin’s lymphomas. Our findings confirm results of previous BCNHL research, indicating that the statistical analyses applied within our computational workflow pipeline are effective at accurately identifying statistically significant genes, splice variants, and pathways with clinical and pathological relevance. Additionally, several of our results are novel, which need additional validation in future experiments. It is likely that at least some of these novel findings were detected due to the ability of our joint secondary analysis to reduce the statistical “noise” produced by outliers from individual studies and increase the biologically-relevant signal. Specifically, our preliminary findings suggest that LUM and 10 other small leucine-rich proteoglycans are significantly differentially expressed in BCNHL, that AL512646.1 is not a pseudo-gene, that APOE, COL1A1, and COL27A1 have significant differentially expressed splice variants in BCNHL, and that BCNHL is strongly reliant on the overexpression of extracellular matrix-associated pathways. The predominant drug prediction results nearly universally targeted extracellular matrix-associated mechanisms and has yielded several promising new potential drug candidates including ocriplasmin and ATN-161. Our random forest biomarker discovery pinpointed three novel biomarker genes not previously associated with BCNHL, YES1, FERMT2, and FAM98B, which show high fidelity in predicting lymphoma presence based on transcriptional levels in B-cells. We believe that additional experiments are needed to validate our results. These findings shed additional light on the underlying intracellular mechanisms of BCNHL and could be used in the development of improved diagnostics and therapeutics to further improve human health. We anticipate that future directions after wet-lab validation could include evaluating FAM98B, FERMT2, and YES1 expression in liquid biopsy as a diagnostic tool, investigating the utility of the predicted drugs against BCNHL, and determining the roles of the genes identifying by our analysis in BCNHL pathology.

## Supplementary Material

Supply

## Figures and Tables

**Table 1:** Study-based origin of samples included in the joint secondary analysis.

Sample Phenotype	Single End or Paired End Reads	GEO Accession #	Relevant Samples
Large B-Cell Lymphoma	Paired End	GSE153437 [9]	25
Diffuse Large B-Cell Lymphoma	Paired End	GSE130751 [10]	63
B-Cell Lymphoma	Single End	GSE110219 [11]	1
Diffuse Large B-Cell Lymphoma	Paired End	GSE95013 [12]	28
Follicular Lymphoma	Paired End	GSE62241 [13,14]	10
Diffuse Large B-Cell Lymphoma	Paired End	GSE50514 [15]	7
Healthy	Single End	GSE110219 [11]	1
Healthy	Paired End	GSE62241 [13,14]	4
Healthy	Paired End	GSE45982 [16,17]	8
Healthy	Single End	GSE92387 [18]	12
Healthy	Paired End	GSE118254 [19]	147
Healthy	Paired End	GSE110999 [20]	16

**Table 2:** Top 20 significant differentially expressed genes between BCNHL and healthy samples.

	Gene Symbol	Ensembl ID	Log_2_ Fold Change (log_2_FC)	FDR-corrected p-value
1	LUM	ENSG00000139329	11.1	1.11 × 10^−145^
2	CXCL9	ENSG00000138755	11	4.31 × 10^−141^
3	C1QC	ENSG00000159189	9.65	2.70 × 10^−132^
4	C1QA	ENSG00000173372	9.54	2.03 × 10^−123^
5	CCL18	ENSG00000278167	10	3.74 × 10^−123^
6	VCAM1	ENSG00000162692	7.58	2.29 × 10^−120^
7	C1QB	ENSG00000173369	9.4	8.19 × 10^−119^
8	APOC1	ENSG00000130208	6.93	8.55 × 10^−117^
9	AL512646.1	ENSG00000203396	−15.6	2.24 × 10^−115^
10	CCL19	ENSG00000172724	8.48	1.27 × 10^−111^
11	SLAMF8	ENSG00000158714	7.77	4.01 × 10^−111^
12	COL3A1	ENSG00000168542	10.1	1.67 × 10^−110^
13	TCIM	ENSG00000176907	8.07	7.86 × 10^−110^
14	RARRES2	ENSG00000106538	7.25	8.21 × 10^−109^
15	CXCL13	ENSG00000156234	8.8	1.72 × 10^−107^
16	SPARCL1	ENSG00000152583	7.24	6.42 × 10^−107^
17	PTGDS	ENSG00000107317	7.69	1.07 × 10^−105^
18	COL1A2	ENSG00000164692	8.33	3.70 × 10^−102^
19	CXXC5	ENSG00000171604	−2.73	3.70 × 10^−102^
20	C1R	ENSG00000159403	4.7	1.41 × 10^−100^

**Table 3:** Novel identification of differential expression of Small Leucine-Rich Proteoglycans (SLRPs) in BCNHL.

SLRP Class	Name	Log_2_ Fold Change	FDR-corrected p-value
Class I	DCN	2.88	1.67 × 10^−41^
	BGN	7.88	3.22 × 10^−95^
	ASPN	3.09	2.27 × 10^−25^
	ECM2	2.1	1.44 × 10^−17^
	ECMX	NP	NP
Class II	FMOD	5.71	3.86 × 10^−61^
	LUM	11.1	1.11 × 10^−145^
	PRELP	0.617	1.54 × 10^−4^
	KERA	NP	NP
	OMD	NP	NP
Class III	EPYC	NP	NP
	OPTC	NP	NP
	OGN	NS	NS
Class IV	CHAD	−3.49	1.33 × 10^−24^
	NYX	NP	NP
	TSKU	1.37	1.62 × 10^−16^
Class V	PODN	1.66	5.70 × 10^−10^
	PODNL1	−1.49	3.15 × 10^−11^

* NS = not significant; NP = not present.

**Table 4:** Top 20 most significant splice variants (sorted by gene).

Gene symbol	Ensembl ID	Lr*	# of Alternate Transcripts	Adjusted P-value
APOE	ENSG00000130203	4470	4	0
COL1A1	ENSG00000108821	1520	12	5.56 × 10^−315^
COL27A1	ENSG00000196739	1060	7	6.71 × 10^−220^
RPL5	ENSG00000122406	1040	10	3.86 × 10^−214^
KLF6	ENSG00000067082	961	6	7.41 × 10^−201^
SRSF6	ENSG00000124193	954	5	1.56 × 10^−200^
CYBRD1	ENSG00000071967	931	6	2.17 × 10^−194^
PLEKHM1P1	ENSG00000214176	924	5	3.78 × 10^−194^
VCP	ENSG00000165280	912	6	2.37 × 10^−190^
DDX6	ENSG00000110367	872	7	8.01 × 10^−181^
THRAP3	ENSG00000054118	846	3	6.63 × 10^−180^
FCGR2B	ENSG00000072694	771	4	1.75 × 10^−162^
CHI3L1	ENSG00000133048	715	4	2.40 × 10^−150^
IFITM3	ENSG00000142089	691	3	2.53 × 10^−146^
ADAM28	ENSG00000042980	719	11	4.56 × 10^−144^
CIB1	ENSG00000185043	662	2	2.04 × 10^−141^
ZNF318	ENSG00000171467	645	3	1.88 × 10^−136^
RPS28	ENSG00000233927	621	3	3.10 × 10^−131^
CCDC124	ENSG00000007080	549	3	1.14 × 10^−115^
ZNF335	ENSG00000198026	545	3	8.02 × 10^−115^

* Lr = likelihood ratio

**Table 5:** Significant differentially modulated signaling pathways include extracellular matrix.

	Name	pSize	NDE	tA	pGFWER	Source Database
1	Integrin signalling pathway	99	86	114.394	2.39 × 10^−5^	Panther
2	Extracellular matrix organization	204	180	80.7398395	3.14 × 10^−5^	Reactome
3	ECM-receptor interaction	70	64	77.659	4.47 × 10^−5^	KEGG
4	Staphylococcus aureus infection	32	29	110.568396	0.000503428	KEGG
5	Complement and coagulation cascades	36	32	37.5461944	0.000674242	KEGG
6	Urokinase-type plasminogen activator (uPA) and uPAR-mediated signaling	28	25	130.821315	0.000740169	NCI
7	Cytokine-cytokine receptor interaction	168	140	98.294	0.000780995	KEGG
8	Focal adhesion	182	150	236.615459	0.001133449	KEGG
9	PI3K-Akt signaling pathway	271	221	260.046197	0.001344307	KEGG
10	Complement cascade	29	27	102.278583	0.001440498	Reactome
11	Systemic lupus erythematosus	17	15	67.4577222	0.001616635	KEGG
12	b cell survival pathway	22	19	26.576	0.00167109	BioCarta
13	Small cell lung cancer	78	64	121.170067	0.001930414	KEGG
14	Integrins in angiogenesis	52	41	146.143424	0.00285984	NCI
15	Olfactory transduction	93	74	−148.8965	0.002966626	KEGG
16	integrin signaling pathway	37	29	77.0156667	0.003336442	BioCarta
17	erk and pi-3 kinase are necessary for collagen binding in corneal epithelia	34	26	166.268917	0.003755165	BioCarta
18	RNA Polymerase I Promoter Clearance	85	72	−40.156	0.004307328	Reactome
19	Initial triggering of complement	15	14	44.508	0.004480759	Reactome
20	RNA Polymerase I Promoter Opening	39	34	−40.907	0.004675938	Reactome
21	RHO GTPases activate PKNs	67	57	39.779	0.004802382	Reactome
22	DNA Damage/Telomere Stress Induced Senescence	61	52	32.7708077	0.004980633	Reactome
23	Creation of C4 and C2 activators	7	7	27.365	0.005633091	Reactome
24	Collagen formation	66	63	26.4272897	0.006045837	Reactome
25	Activated PKN1 stimulates transcription of AR (androgen receptor) regulated genes KLK2 and KLK3	41	35	39.094	0.006675281	Reactome
26	MET activates PTK2 signaling	18	16	63.573	0.007079287	Reactome
27	Collagen degradation	17	15	131.8905	0.008037505	Reactome
28	MET promotes cell motility	28	24	97.5445	0.00808298	Reactome
29	Regulation of IGF Activity by IGFBP	11	10	25.958725	0.008404989	Reactome
30	Classical antibody-mediated complement activation	5	5	27.354	0.008619298	Reactome
31	Serotonin Neurotransmitter Release Cycle	11	9	−13.301889	0.015608196	Reactome
32	Class A/1 (Rhodopsin-like receptors)	81	77	5.908	0.026176599	Reactome
33	Peptide ligand-binding receptors	79	75	5.84	0.040928099	Reactome

* Abbreviations: psize = number of genes in pathway. NDE = number of genes from pathway which were differentially expressed. tA = measure of change between healthy and lymphoma expression; directionality indicates up- or down-modulation. pGFWER = p-value with adjustments appropriate to a multiplexed interaction network [31].

**Table 6:** Predicted BCNHL drugs based on signaling pathways.

	Drug Name	Drug ID	Significant Pathways Targeted	Is FDA Approved for Human Use in > 1 Indication	Highest Clinical Trial Phase	Has Been Withdrawn
1	OCRIPLASMIN	CHEMBL2095222	13	TRUE	4	FALSE
2	ATN-161	CHEMBL4297456	10	FALSE	2	FALSE
3	DOXYCYCLINE	CHEMBL1200699	10	TRUE	4	FALSE
4	DOXYCYCLINE	CHEMBL1433	10	TRUE	4	FALSE
5	AS-1409	CHEMBL2109413	9	FALSE	1	FALSE
6	COLLAGENASE CLOSTRIDIUM HISTOLYTICUM	CHEMBL2108709	9	TRUE	4	FALSE
7	FIRATEGRAST	CHEMBL2104967	9	FALSE	2	FALSE
8	L19IL2	CHEMBL2109608	9	FALSE	3	FALSE
9	L19SIP 131I	CHEMBL2109412	9	FALSE	2	FALSE
10	L19TNFA	CHEMBL2109589	9	FALSE	2	FALSE
11	VOLOCIXIMAB	CHEMBL2108061	9	FALSE	3	FALSE
12	ABITUZUMAB	CHEMBL2109621	8	FALSE	2	FALSE
13	AL-78898A	CHEMBL4594457	8	FALSE	2	FALSE
14	CILENGITIDE	CHEMBL429876	8	FALSE	3	FALSE
15	EPTIFIBATIDE	CHEMBL1174	8	TRUE	4	FALSE
16	ETARACIZUMAB	CHEMBL1743014	8	FALSE	2	FALSE
17	HUMAN C1-ESTERASE INHIBITOR	CHEMBL4297549	8	TRUE	4	FALSE
18	INTETUMUMAB	CHEMBL1743032	8	FALSE	2	FALSE
19	PEGCETACOPLAN	CHEMBL4298211	8	FALSE	3	FALSE
20	STX-100	CHEMBL2109623	8	FALSE	2	FALSE

**Table 7:** BCNHL biomarkers predicted from gene expression using machine learning.

Gene Symbol	Mean Gini Impurity Decrease	edgeR Log_2_ Fold Change	edgeR FDR P-value	Disease Status	Mean (Read Counts)	Standard Deviation (Read Counts)	Median (Read Counts)
YES1	0.77	2.38	1.98x10^−38^	Lymphoma	1151.756	1246.946	629
				Healthy	38.87234	66.01043	11
FAM98B	0.68	1.58	1.48x10^−60^	Lymphoma	1797.452	1174.797	1456
				Healthy	248.4202	954.1375	32
FERMT2	0.67	2.83	1.46x10^−38^	Lymphoma	1246.993	1200.669	841
				Healthy	32.46809	73.10299	4

## Abbreviations:

BCNHL: B-cell Non-Hodgkin Lymphoma
RNA-seq: RNA-sequencing
NCBI: National Center for Biotechnology Information
GEO: Gene Expression Omnibus
SRA: Sequence Read Archive
ARMOR: Automated Reproducible MOdular workflow for preprocessing and differential analysis of RNA-seq data
SPIA: Signaling Pathway Impact Analysis
GO: Gene Ontology
AUC: Area Under The Curve
DEGs: Differentially Expressed Genes
APOC1: Apolipoprotein C1
log_2_FC: log_2_ Fold Change
VCAM1: Vascular Adhesion Molecule 1
CCL18: C-C Motif Chemokine Ligand 18
CXCL9: C-X-C Motif Chemokine Ligand 9
LUM: Lumican
SLRPs: Small Leucine-Rich Proteoglycans
NS: Not Significant
NP: Not Present
C1QA: Complement C1q A chain
C1QB: Complement C1q B chain
C1QC: Complement C1q C chain
APOE: Apolipoprotein E
Lr: Likelihood ratio
COL1A1: Collagen type I Alpha 1 chain
COL27A1: Collagen type XXVII alpha 1 chain
psize: Number of Genes in a Pathway
NDE: Number of Differentially-Expressed Genes in Pathway
YES1: YES Proto-Oncogene 1 Src Family Tyrosine Kinase
FERMT2: FERM Domain Containing Kindlin 2
FAM98B: Family with Sequence Similarity 98 Member B
ROC: Receiver Operator Curve
DLBCL: Diffuse Large B-Cell Lymphoma
CTNNB1: Beta-Catenin/Beta Catenin
JNK: Janus Kinase
IGF: Insulin-like Growth Factor
IGFBP: Insulin-like Growth Factor Binding Protein
