# Supplementary figures and images for: Joint Secondary Transcriptomic Analysis of Non-Hodgkin’s B-Cell Lymphomas Predicts Reliance on Pathways Associated with the Extracellular Matrix and Robust Diagnostic Biomarkers
Source: J Bioinform Syst Biol. Author manuscript; Available in PMC 2023 Mar 2. (PMC9980876; doi:10.26502/jbsb.5107040)

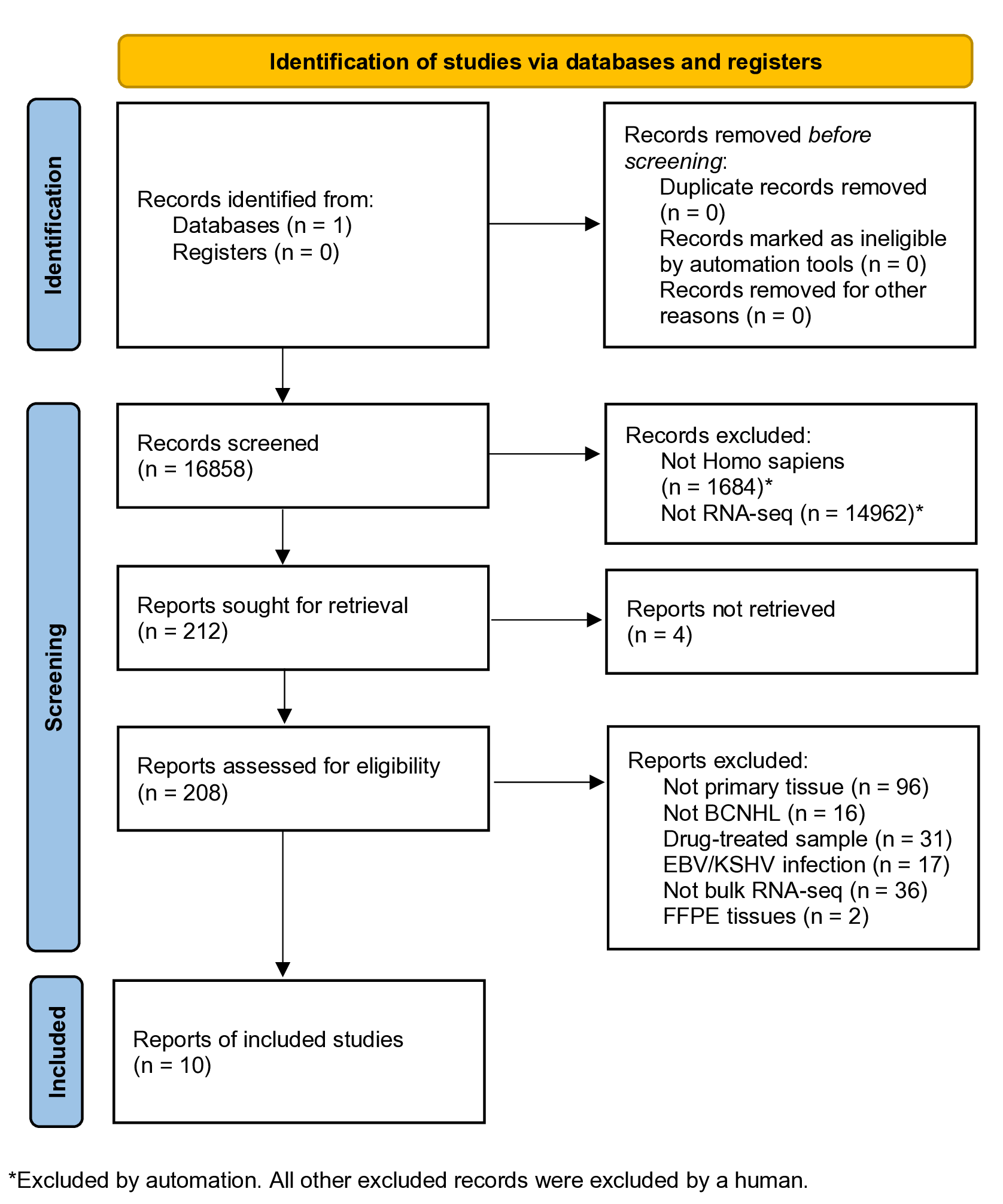

Supplement: Supply [file NIHMS1870914-supplement-Supply.zip › SUPPLEMENTARY_MATERIALS_Lymphoma_transcr_NR-S/Fig_1.tiff]

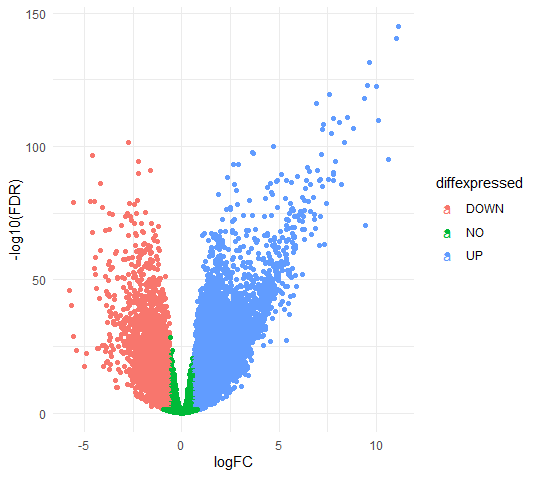

Supplement: Supply [file NIHMS1870914-supplement-Supply.zip › SUPPLEMENTARY_MATERIALS_Lymphoma_transcr_NR-S/Fig_2.tiff]

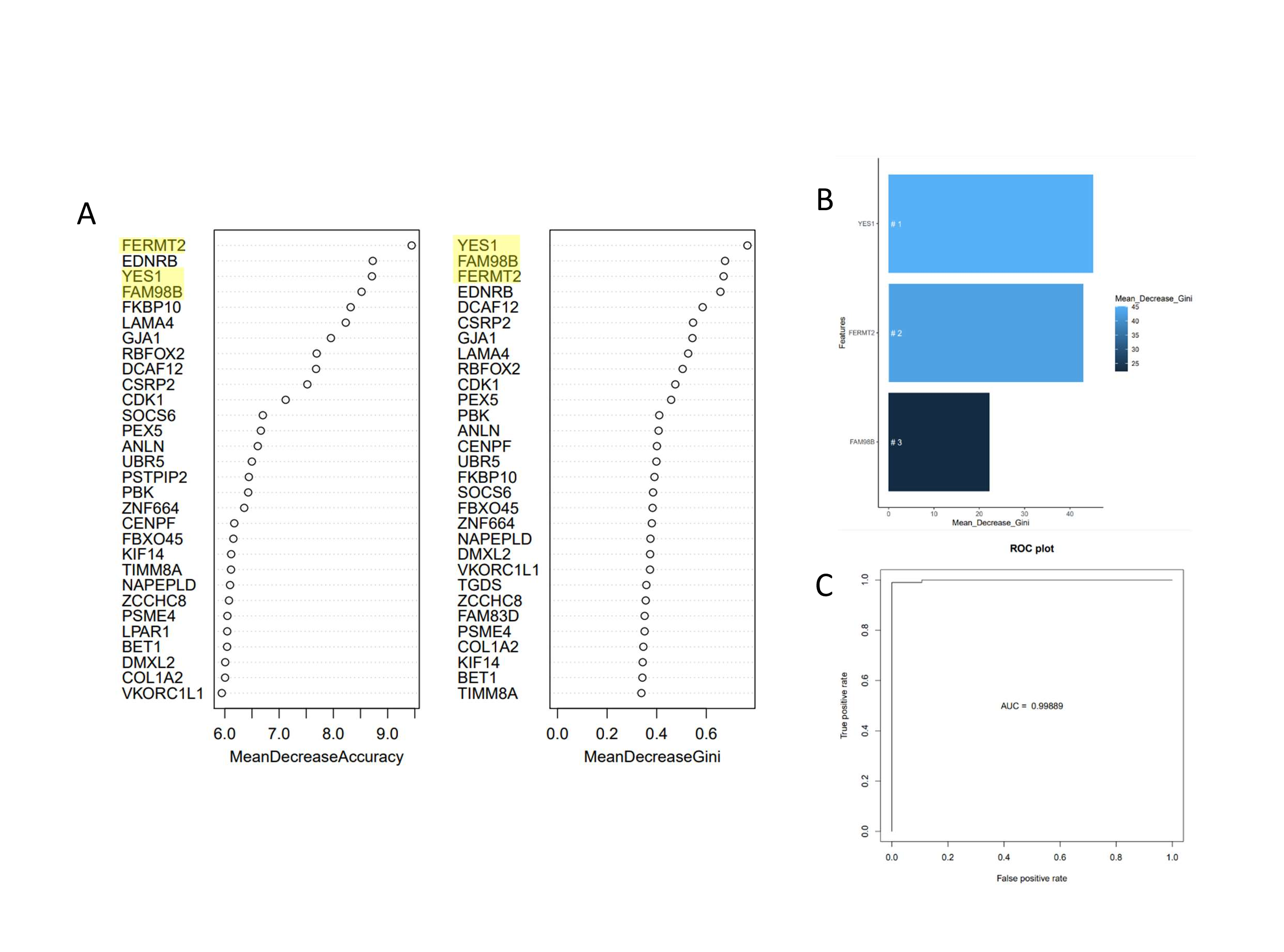

Supplement: Supply [file NIHMS1870914-supplement-Supply.zip › SUPPLEMENTARY_MATERIALS_Lymphoma_transcr_NR-S/Fig_3.tiff]

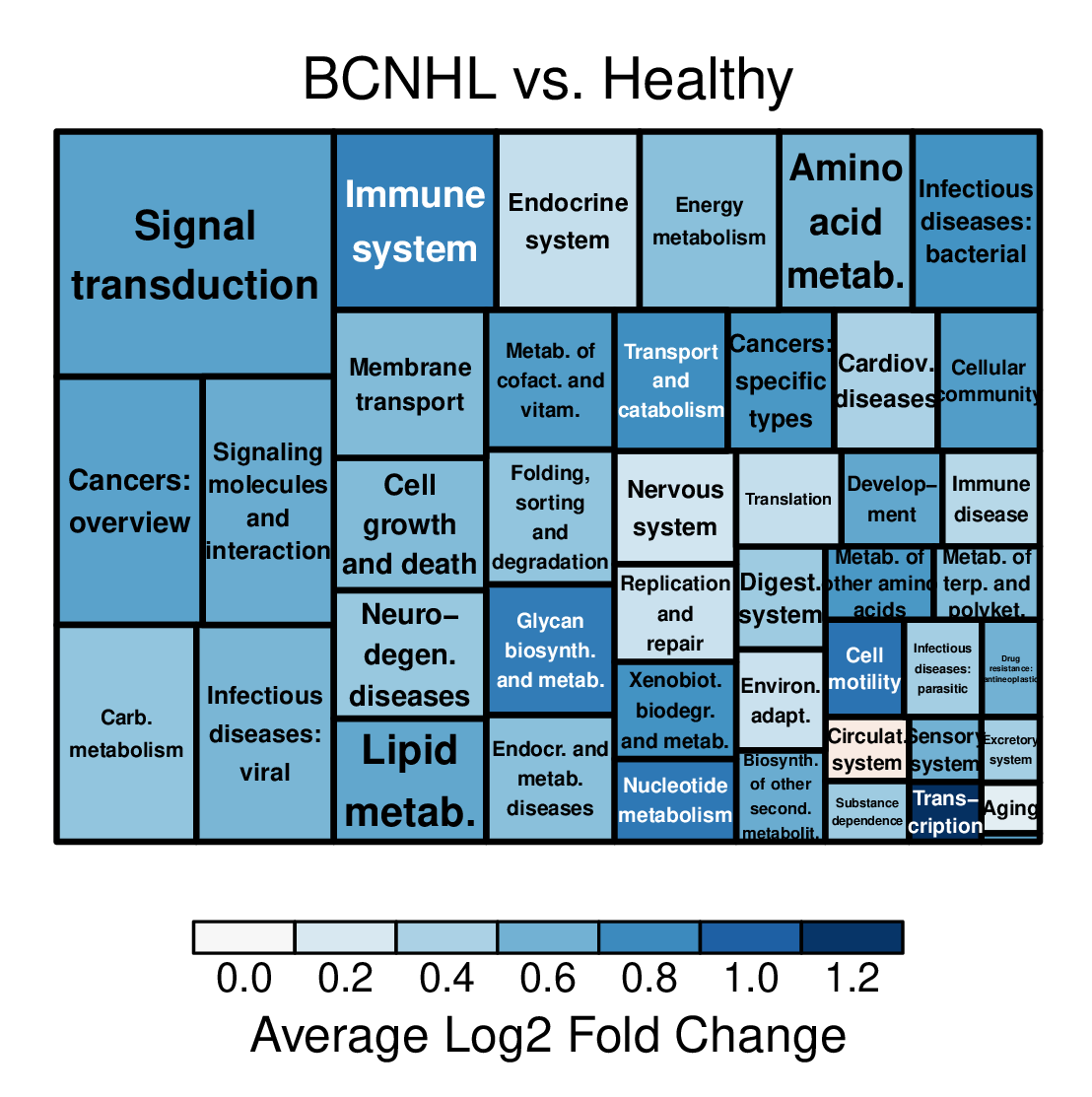

Supplement: Supply [file NIHMS1870914-supplement-Supply.zip › SUPPLEMENTARY_MATERIALS_Lymphoma_transcr_NR-S/S3_File.tiff]
